# An anticholinergic burden score for German prescribers: score development

**DOI:** 10.1186/s12877-018-0929-6

**Published:** 2018-10-11

**Authors:** Esther Katharina Kiesel, Yvonne Marina Hopf, Michael Drey

**Affiliations:** 1 Department of Pharmacy, University Hospital, LMU Munich, Munich, Germany; 2 Department of Medicine IV, University Hospital, LMU Munich, Munich, Germany

**Keywords:** Anticholinergic, Geriatrics, Anticholinergic scales, Anticholinergic burden, Germany, Expert opinion, Potentially inappropriate medicine

## Abstract

**Background:**

Anticholinergic drugs put elderly patients at a higher risk for falls, cognitive decline, and delirium as well as peripheral adverse reactions like dry mouth or constipation. Prescribers are often unaware of the drug-based anticholinergic burden (ACB) of their patients. This study aimed to develop an anticholinergic burden score for drugs licensed in Germany to be used by clinicians at prescribing level.

**Methods:**

A systematic literature search in pubmed assessed previously published ACB tools. Quantitative grading scores were extracted, reduced to drugs available in Germany, and reevaluated by expert discussion. Drugs were scored as having no, weak, moderate, or strong anticholinergic effects. Further drugs were identified in clinical routine and included as well.

**Results:**

The literature search identified 692 different drugs, with 548 drugs available in Germany. After exclusion of drugs due to no systemic effect or scoring of drug combinations (*n* = 67) and evaluation of 26 additional identified drugs in clinical routine, 504 drugs were scored. Of those, 356 drugs were categorised as having no, 104 drugs were scored as weak, 18 as moderate and 29 as having strong anticholinergic effects.

**Conclusions:**

The newly created ACB score for drugs authorized in Germany can be used in daily clinical practice to reduce potentially inappropriate medications for elderly patients. Further clinical studies investigating its effect on reducing anticholinergic side effects are necessary for validation.

**Electronic supplementary material:**

The online version of this article (10.1186/s12877-018-0929-6) contains supplementary material, which is available to authorized users.

## Background

Studies show that over 50% of elderly patients take five or more drugs, both prescription and over-the-counter [1]. A cross-sectional study in Germany revealed further that 62% of people aged 65 or older suffer from multimorbidity [2]. This combination of multimorbidity and polypharmacy leads to a higher risk for drug-drug interactions and adverse drug reactions (ADRs) [3, 4]. Hence, the use of drugs should be considered carefully in geriatric patients. Part of this consideration should be to avoid potentially inappropriate medications.

Drugs with anticholinergic properties are part of inappropriate medications for geriatric patients [5, 6]. Anticholinergic activity of multiple drugs add up to the so-called anticholinergic burden (ACB). Older patients are more prone to anticholinergic side effects than younger patients [5, 6]. Due to reduced metabolic capacity and slowed elimination in older patients, drugs are eliminated slower, in addition to age-related reductions in cholinergic transmissions [5, 6]. The permeability of the blood-brain-barrier increases which leads to a higher drug concentration in the central nervous system [7]. Central nervous ADRs may especially increase the risk of falls, e.g. blurred vision, confusion, or tremors [6]. More severe anticholinergic side effects are tachyarrhythmia, hallucinations, delirium, and cognitive impairment [6]. Other ADRs like dry mouth, constipation, and urinary retention might reduce the quality of life [6]. Several reviews indicate a higher risk for falls, cognitive decline and delirium with an increased ACB of older patients [8–12].

Despite these apparent risks it is estimated that approximately 50% of elderly people take anticholinergic medications [13, 14]. Qualitative studies demonstrated that very few prescribers are aware of the anticholinergic properties of drugs and of the nature of anticholinergic side effects [15]. Even if they know about the negative impact of anticholinergic medications, they hesitate to deprescribe or change those drugs as they do not feel responsible or lack time, knowledge, and resources [16]. In clinical practice there is rarely a registration, documentation and/or conscious reduction of the ACB [15, 17].

Worldwide studies identified over 100 different drugs as having anticholinergic properties [9, 10, 18–33]. Depending on study population, method, and setting, there are different drug lists and different scales to calculate the ACB [18, 19]. The variety of scales and systems complicates the implementation in practice.

Therefore, the current study aimed to develop an anticholinergic burden score specifically for the German healthcare system. This, in turn, can help German prescribers identify and reduce drugs with anticholinergic properties in geriatric patients in order to facilitate easy application in the daily clinical setting. To our knowledge a specific ACB score for Germany is not yet available.

## Methods

**Fig. 1:**
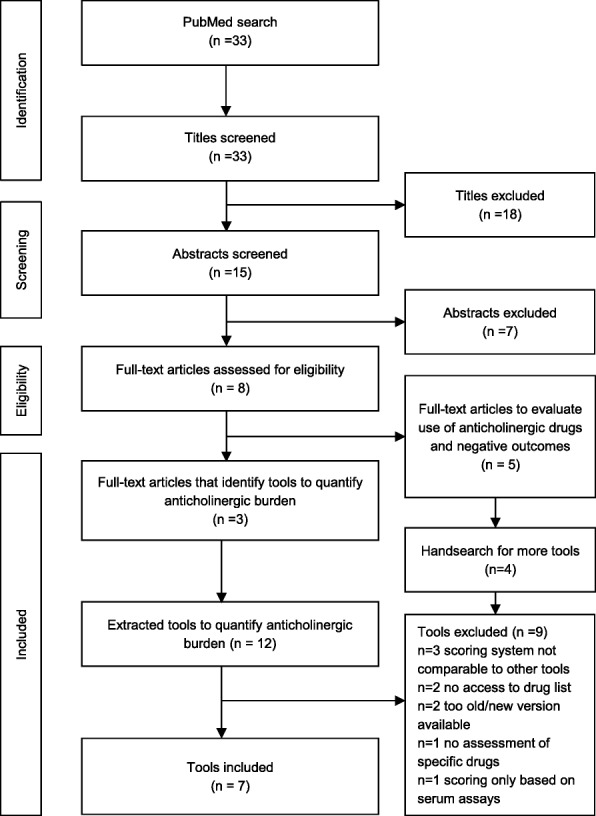
Identification of tools

To identify appropriate tools, PubMed was searched for systematic reviews on tools to quantify anticholinergic drug burden. The search terms were “review AND anticholinergic burden AND (scale OR list OR tool)” without a date limitation. The search was conducted on December 1, 2016. The search and identification process is presented in Fig. 1. Articles were excluded if they were not systematic reviews on tools to quantify anticholinergic drug burden or the language was not English. Three systematic reviews [11, 18, 34] were included identifying 12 tools to quantify anticholinergic burden [10, 20–26, 32, 33, 35, 36]. Tools were excluded because the scoring system was not comparable to the other tools [33, 36], the tool was outdated, [35] there was an updated version published [32], or the scoring was solely based on serum assays [25]. Literature reviews and meta-analysis failed to show an association of serum anticholinergic activity and anticholinergic effects [37], whereas there is an association shown for anticholinergic drug scores mainly based on expert opinion [8–12, 27]. To avoid missing relevant tools, the excluded articles were reviewed regarding more tools as they all evaluated the association of anticholinergic drugs and negative outcomes in patients [9, 19, 27, 38, 39]. These reviews identified four further tools, all ineligible for inclusion because (1) it was impossible to access the drug list despite contacting the authors [28, 31], (2) the scoring system was not comparable to other drug lists [29], and (3) the study assessed the overall medication of patients not specific drugs [30]. The included anticholinergic drug lists were summarized and reduced to drugs available in Germany [10, 20–24, 26]. For Boustani et al. an updated version was included [10, 40].

**Fig. 2:**
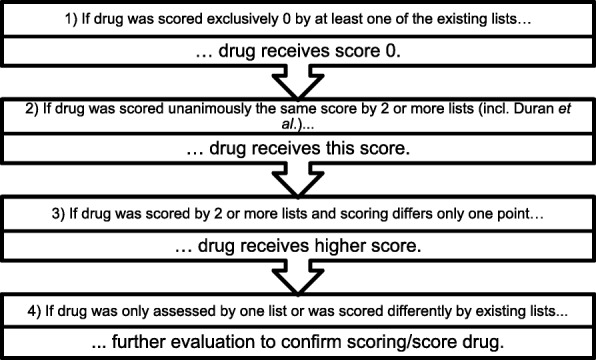
Algorithm for consistent scoring

The approach to merge the identified scales was similar to the approach of Duran et al. [18]. For all scales, quantitative grading scores proposed by the authors were extracted. Most lists used scores ranging from 0 to 3, one was modified according to Duran et al. so that its 0–4 scale was comparable to 0–3 scales [18, 24]. Topical, ophthalmic, otic and nasal drugs were excluded, while oral, parenteral, inhalative and transdermal drugs were included as these are more likely to show systemic effects [41]. As these lists have only low to moderate concordance, the algorithm depicted in Fig. 2 was used to get a consistent scoring.

Where further evaluation was needed as existing lists scored them differently or only one of the scores evaluated that specific drug., one researcher (EK) looked at the mechanism of action and the ADRs as reported in the German Summary of Product Characteristics and DRUGDEX® (expert-reviewed database for detailed drug information) to assess the anticholinergic properties of the respective drug. This assessment, the existing scores and the scoring by Duran et al. [18] were discussed in a multidisciplinary team of one geriatrician and two clinical pharmacists. This discussion led to a final score. Each drug was coded according to the Anatomical, Therapeutic and Chemical (ATC) Classification from the World Health Organization. If there were discrepancies in rating within the same drug class, we reconsidered some ratings as well.

**Fig. 3:**
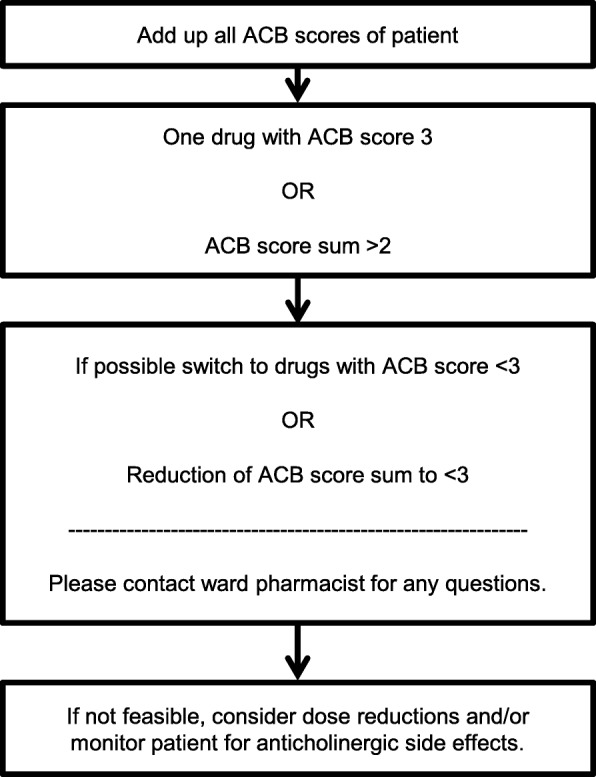
Recommendation for prescribers on pocket-card based on Boustani et al. [10]

In order to tailor the ACB list to the clinical setting a retrospective evaluation of admission and discharge medication on an acute geriatric ward identified a range of further drugs. Consecutive patients for two 6-week-periods were included, which meant the admission and discharge medication of 34 patients was evaluated. The patients were prescribed a total of 235 drugs at admission and 276 drugs at discharge. All drugs thus identified that were not mentioned in the reviewed scales were evaluated for their anticholinergic properties as described above. Based on our final anticholinergic drug list, we designed a pocket-sized guideline for prescribers with information on anticholinergic drugs, anticholinergic side effects and recommendations how to assess and handle the ACB of patients on ward. The recommendation was based on the approach of Boustani et al. and is presented in Fig. 3 [10].

The anticholinergic scores of all drugs used by a patient were calculated in total. If one drug scored 3 or the patient had a summated score of 3 or higher, it was recommended to switch to alternative drugs with a lesser ACB score in order to reduce the summated score to < 3 [10]. If discontinuation or switching was not possible, a dose reduction and/or monitoring for ADRs were advised. Through the upper limit of score 3, the suggested ceiling effect of the ACB is included [19]. This means that at a high ACB, drug effects are assumed to reach a plateau even when the number of anticholinergic drugs further increases [19].

## Results

The summary of existing anticholinergic drug lists [20–24, 26, 40] resulted in a list of 692 different drugs with 548 available in Germany (for excluded, international drugs see Additional file 1: Table S1). In total, 67 drugs were excluded because they were rated for topical, nasal, ophthalmic, or otic application only, or were drug combinations (Additional file 1: Table S2).

In terms of effect, 334 of the drugs were categorised as having no anticholinergic effects (ACB score = 0), 77 of the drugs were scored as displaying weak anticholinergic effects (ACB score = 1), 10 as moderate anticholinergic effects (ACB score = 2) and 27 as strong anticholinergic effects (ACB score = 3). These anticholinergic drugs are displayed in Table 1. Drugs rated as having no anticholinergic effects are available in the supplementary material.

**Table 1:** Anticholinergic drugs with concordant ratings of sores

Drug	ATC-Code	Carnahan 2006 USA [20]	Ancelin 2006 France [21]	Rudolph 2008 USA [22]	Han 2008 USA [23]	Ehrt 2010 Norway modified [24]	Sittironnarit 2011 Australia [26]	Boustani 2012 USA [40]	Duran 2013 [18]
Weak anticholinergic effects ACB score 1									
Ampicillin	J01CA01	1							0 or 1
Aripiprazole	N05AX12						0	1	
Atenolol	C07AB03	0			1	0	0	1	0
Azathioprine	L04AX01	1							0 or 1
Benazepril	C09AA07	0			1				0 or 1
Betaxolol	C07AB05				1		0		0 or 1
Bisacodyl	A06AB02	0					1		0 or 1
Bromocriptine	N04 BC01	1				0			weak(1–2)
Bupropion	N06AX12	0			1			1	0 or 1
Captopril	C09AA01	1				0	0	1	0 or 1
Celecoxib	M01AH01	0					1		0 or 1
Chlordiazepoxide	N05BA02	1			1				weak(1–2)
Chlorthalidone	C03BA04	1					0	1	0 or 1
Ciclosporin	L04 AD01	1							0 or 1
Citalopram	N06AB04	0				1	1		weak(1–2)
Clindamycin	J01FF01	1							0 or 1
Clonazepam	N03AE01	1					1		weak(1–2)
Dexamethasone	H02AB02	1					0		0 or 1
Dextromethorphan	R05DA09	0			1				0 or 1
Diazepam	N05BA01	1			1	1	1	1	weak(1–2)
Digitoxin	C01AA04	1				1			weak(1–2)
Diltiazem	C08DB01	1					0		0 or 1
Dipyridamole	B01AC07	1				0		1	0 or 1
Domperidone	A03FA03						1		weak(1–2)
Entacapone	N04BX02	0		1					weak(1–2)
Escitalopram	N06AB10	0					1		0 or 1
Famotidine	A02BA03	1					0		0 or 1
Fentanyl	N02AB03	1						1	weak(1–2)
Flunitrazepam	N05CD03					1			0 or 1
Fluoxetine	N06AB03	1			1	1	1		weak(1–2)
Flurazepam	N05CD01	1							0 or 1
Fluvoxamine	N06AB08	1				1	1	1	weak(1–2)
Gentamicin	J01GB03	1							0 or 1
Guaifenesin	R05CA03	0			1				0 or 1
Hydralazine	C02DB02	1					0	1	0 or 1
Hydrocortisone	H02AB09	1						1	0 or 1
Isosorbide dinitrate	C01DA08	1				0	0		0 or 1
Isosorbide mononitrate	C01DA14	1					0		0 or 1
Ketorolac	M01AB15				1				weak(1–2)
Lansoprazole	A02BC03	0				1	0		0 or 1
Levodopa	N04BA01	0		1	1	0	0 or 1		0 or 1
Lithium	N05AN01	0					1		weak(1–2)
Lorazepam	N05BA06	1							0 or 1
Metformin	A10BA02	0					1		0 or 1
Methotrexate	L04AX03	0					1		0 or 1
Methylprednisolone	H02AB04	1					0		0 or 1
Metoprolol	C07AB02	0			1	0	0	1	0
Midazolam	N05CD08	1							0 or 1
Mirtazapine	N06AX11	0		1					weak(1–2)
Morphine	N02AA01	1			1			1	weak(1–2)
Naratriptan	N02CC02						1		0 or 1
Nifedipine	C08CA05	1				0	0	1	0
Oxazepam	N05BA04	1				0	1		0 or 1
Oxycodone	N02AA05	1			1		1		weak(1–2)
Pancuronium	M03 AC01	1							0 or 1
Phenobarbital	N03AA02	0			1	1			0 or 1
Piperacillin	J01CA12	1							0 or 1
Pramipexole	N04 BC05	0		1		0			0 or 1
Prednisolone	A07EA01	1				0	0		0 or 1
Prednisone	A07EA03	1						1	
Quinidine	C01BA01	0						1	
Risperidone	N05AX08	0		1	1		1	1	weak(1–2)
Selegiline	N04BD01	0		1		0			0 or 1
Sertraline	N06AB06	1			1	0	0		0
Sumatriptan	N02CC01						1		0 or 1
Temazepam	N05CD07	1					1		weak(1–2)
Trandolapril	C09AA10	0			1		0		0 or 1
Trazodone	N06AX05	0		1	1			1	weak(1–2)
Triamcinolone	H02AB08	1					0		0 or 1
Triamterene	C03DB02	1					0	1	0 or 1
Triazolam	N05CD05	1			1				weak(1–2)
Valproic acid	N03AG01	1					0		0 or 1
Vancomycin	J01XA01	1							0 or 1
Venlafaxine	N06AX16	0			1	0	1	1	0
Warfarin	B01AA03	1				0	0	1	0
Ziprasidone	N05AE04			1					0 or 1
Zolmitriptan	N02CC03						1		0 or 1
Moderate anticholinergic effects ACB score 2									
Amantadine	N04BB01	1		2				2	weak(1–2)
Cimetidine	A02BA01	2		2				1	weak(1–2)
Loperamide	A07DA03	1		2	1		1	1	weak(1–2)
Loxapine	N05AH01	2						2	weak(1–2)
Methadone	N07 BC02				2				weak(1–2)
Oxcarbazepine	N03AF02	2						2	weak(1–2)
Pimozide	N05AG02	2						2	weak(1–2)
Ranitidine	A02BA02	2		1	2	1	1	1	weak(1–2)
Theophylline	R03DA04	1	2			1	2	1	weak(1–2)
Tramadol	N02AX02	1			2		2		weak(1–2)
Strong anticholinergic effects ACB score 3									
Amitriptyline	N06AA09	3	3	3	3	3	3	3	Strong(3)
Atropine	A03BA01	3		3	3		3	3	Strong(3)
Chlorpheniramine	R06AB04	3	3	3	3		3	3	Strong(3)
Clemastine	R06AA04	3						3	Strong(3)
Clomipramine	N06AA04	3	3					3	Strong(3)
Clozapine	N05AH02	3		2		3		3	Strong(3)
Cyproheptadine	R06AX02	2		3			3	2	Strong(3)
Darifenacin	G04BD10	3						3	Strong(3)
Dimenhydrinate	A04AB02	3						3	Strong(3)
Diphenhydramine	A04AB05	3		3	3			3	Strong(3)
Doxepin	N06AA12	3			3	3	3	3	Strong(3)
Flavoxate	G04BD02	3						3	Strong(3)
Hydroxyzine	N05BB01	3	3	3				3	Strong(3)
Imipramine	N06AA02	3	3	3	3		3	3	Strong(3)
Levomepromazine	N05AA02	2	3					2	Strong(3)
Nortriptyline	N06AA10	3		2	3	2		3	Strong(3)
Orphenadrine	N04AB02	3	3			3		3	Strong(3)
Oxybutynin	G04BD04	3	3	3		3	2	3	Strong(3)
Procyclidine	N04AA04	3							Strong(3)
Scopolamine	A04AD01	3			3			3	Strong(3)
Thioridazine	N05 AC02	3		3	3	3		3	Strong(3)
Tizanidine	M03BX02			3					Strong(3)
Tolterodine	G04BD07	3		2	3		3	3	Strong(3)
Trihexyphenidyl	N04AA01	3	3		3	3		3	Strong(3)
Trimipramine	N06AA06	3	3			3		3	Strong(3)

Drugs are sorted by their assigned score and then alphabetical

Further evaluation was required for 35 drugs. After consideration of adverse drug reactions and mechanism of action, we scored one drug with no anticholinergic effects (ACB score = 0), 22 drugs with weak anticholinergic effects (ACB score = 1), eight as moderate anticholinergic effects (ACB score = 2), and four as having strong anticholinergic effects (ACB score = 3). See Table 2 for specific drugs.

**Table 2:** Scoring of drugs with discrepant ratings (=ratings differed more than 1 score) or only one previous scoring

Drug	ATC-Code	Carnahan 2006 USA [20]	Ancelin 2006 France [21]	Rudolph 2008 USA [22]	Han 2008 USA [23]	Ehrt 2010 Norway modified [24]	Sittironnarit 2011 Australia [26]	Boustani 2012 USA [40]	Duran 2013 [18]
No anticholinergic effects ACB score 0									
Colchicine	M04 AC01	0	3				0	1	discrepant
Weak anticholinergic effects ACB score 1									
Alprazolam	N05BA12	1	3		1		1	1	discrepant
Asenapine	N05AH05							1	
Baclofen	M03BX01	0		2	2				1 or 2
Cetirizine	R06AE07	0		2	2		2	1	1 or 2
Clorazepate	N05BA05	1	3					1	discrepant
Codeine	R05DA04	1	2		1	0	1	1	1 or 2
Desloratadine	R06AX27							1	
Digoxin	C01AA05	1	3			1	1	1	discrepant
Doxylamine	R06AA09						0	3	
Fexofenadine	R06AX26	0			2		2	0	1 or 2
Fluphenazine	N05AB02	1		3			3		3
Furosemide	C03CA01	1	3			1	0	1	discrepant
Ipratropium inhalative	R03BB01	0				3			3
Levocetirizine	R06AE09							1	
Loratadine	R06AX13	0		2	1		1	1	1 or 2
Methocarbamol	M03BA03			1	1			3	1 or 2
Metoclopramide	A03FA01	0		1	3	0	1		discrepant
Paliperidone	N05AX13							1	
Perphenazine	N05AB03	1		3	2	0		3	discrepant
Promethazine	R06AD02	3		3			0	3	3
Pseudoephedrine	R01BA02	0					2		0 oder 1
Tiotropium inhalative	R03BB04						0		
Moderate anticholinergic effects ACB score 2									
Carbamazepine	N03AF01	2			1	0	0	2	1 or 2
Haloperidol	N05 AD01	0		1		0	2	1	1 or 2
Maprotiline	N06AA21		3						discrepant
Pethidine	N02AB02	2				0		2	1 or 2
Olanzapine	N05AH03	1		2	1	2		3	1 or 2
Opipramol	N06AA05		3						discrepant
Paroxetine	N06AB05	1		1	2	2	2	3	1 or 2
Quetiapine	N05AH04	0		1	2	1		3	1 or 2
Strong anticholinergic effects ACB score 3									
Fesoterodine	G04BD11							3	
Propiverine	G04BD06							3	
Solifenacin	G04BD08						0	3	
Trospium	G04BD09							3	

Drugs are sorted first by their assigned score and then alphabetical

During the retrospective evaluation, 26 drugs were identified that were not yet scored by these already existing scores. Parallel to drugs with inconsistent scores, the adverse drug reactions and the mechanism of action were reviewed and the drugs discussed by three researchers to score the drugs. Five drugs were scored as weak anticholinergic effects (ACB score = 1) and 21 drugs were categorised as having no anticholinergic effects (ACB score = 0). See Table 3 for specific drugs.

**Table 3:** Drugs added during retrospective evaluation

Drug	ATC-Code
No anticholinergic effects ACB score 0	
Agomelatine	N06AX22
Apixaban	B01FAF02
Colecalciferol	A11CC05
Dabigatran	B01AE07
Dulaglutide	A10BJ05
Edoxaban	B01AF03
Empagliflozin	A10BK03
Fenoterol inhalative	R03AC04
Formoterol inhalative	R03AC13
Metamizole	N02BB02
Saccharomyces boulardii	A07FA02
Phenprocoumon	B01AA04
Pipamperone	N05 AD05
Piritramide	N02 AC03
Rivaroxaban	B01AF01
Sevelamer	V03AE02
Sitagliptin	A10BH01
Teriparatide	H05AA02
Thiamazole	H03BB02
Tilidine/Naloxone	N02AX51
Vemurafenib	L01XE15
Weak anticholinergic effects ACB score 1	
Aclidinium inhalative	R03BB05
Dimetindene	R06AB03
Etoricoxib	M01AH05
Glycopyrronium inhalative	R03BB06
Rotigotine patch	N04 BC09

Drugs are sorted by their assigned score and then alphabetical

Table 4 shows all drugs scored sorted by their score. Additional file 1: Table S3 shows all drugs scored 0.

**Table 4:** Overview of all drugs scored. Caution: This list does not contain necessarily all drugs with anticholinergic properties

ACB score = 1			ACB score = 2	ACB score = 3
Aclidinium^inh^ Alprazolam Ampicillin Aripiprazole Asenapine Atenolol Azathioprine Baclofen Benazepril Betaxolol Bisacodyl Bromocriptine Bupropion Captopril Celecoxib Cetirizine Chlordiazepoxide Chlorthalidone Ciclosporin Citalopram Clindamycin Clonazepam Clorazepate Codeine Desloratadine Dexamethasone Dextromethorphan Diazepam Digitoxin Digoxin Diltiazem Dimetindene Dipyridamole Domperidone Doxylamine	Entacapone Escitalopram Etoricoxib Famotidine Fentanyl Fexofenadine Flunitrazepam Fluoxetine Fluphenazine Flurazepam Fluvoxamine Furosemide Gentamicin Glycopyrronium^inh^ Guaifenesin Hydralazine Hydrocortisone Ipratropium^inh^ Isosorbide dinitrate Isosorbide mononitrate Ketorolac Lansoprazole Levocetirizine Levodopa Lithium Loratadine Lorazepam Metformin Methocarbamol Methotrexate Methylprednisolone Metoclopramide Metoprolol Midazolam	Mirtazapine Morphine Naratriptan Nifedipine Oxazepam Oxycodone Paliperidone Pancuronium Perphenazine Phenobarbital Piperacillin Pramipexole Prednisolone Prednisone Promethazine Pseudoephedrine Quinidine Risperidone Rotigotine patch Selegiline Sertraline Sumatriptan Temazepam Tiotropium^inh^ Trandolapril Trazodone Triamcinolone Triamterene Triazolam Valproic acid Vancomycin Venlafaxine Warfarin Ziprasidone Zolmitriptan	Amantadine Carbamazepine Cimetidine Haloperidol Loperamide Loxapine Maprotiline Methadone Olanzapine Opipramol Oxcarbazepine Paroxetine Pethidine Pimozide Quetiapine Ranitidine Theophylline Tramadol	Amitriptyline Atropine Chlorpheniramine Clemastine Clomipramine Clozapine Cyproheptadine Darifenacin Dimenhydrinate Diphenhydramine Doxepin Fesoterodine Flavoxate Hydroxyzine Imipramine Levomepromazine Nortriptyline Orphenadrine Oxybutynin Procyclidine Propiverine Scopolamine Solifenacin Thioridazine Tizanidine Tolterodine Trihexyphenidyl Trimipramine Trospium

^inh^inhalative

## Discussion

To our knowledge, this is the first ACB score developed especially for prescribers in Germany. There have been similar international publications [18, 27]. The drugs most commonly used in Germany differ from other countries especially England, USA, and Australia, where many published studies on anticholinergic drugs were conducted. Our ACB score did not only summarize existing scores but re-evaluated the drugs, especially those with discrepancies, and reduced the list to those authorized in Germany. This saves valuable time and effort for clinicians trying to evaluate anticholinergic burden in patients.

The scores used were identified via a systematic literature search in pubmed. This systematic approach should ensure a replicable and complete choice of peer-reviewed and published ACB scores, although it was not a systematic literature review conducted in different databases. All included scores were previously validated. Drug evaluation was based on expert opinion which was previously preferred to measuring serum assays [18, 27]. This expert review of the drugs by three different people (one geriatrician and two clinical pharmacists) based on clinical experience and literature data on method of action and ADRs strengthens the development of this score. Scoring was confirmed by the expert committee not only for drugs with discrepancies, but also for drugs that were only scored by one of the existing scores. Being rated by only one score is not necessarily a limitation as the individual selection of drugs is always depending on the country, the setting, and other specifics of the score development.

As our score is based on previously published ACB scores and drugs within our hospital during a retrospective evaluation, we do not claim this list to be comprehensive. There are over 2000 drugs approved in Germany, so there are potentially more anticholinergic drugs not yet considered in this list. There were few drugs found in our retrospective evaluation that had not been rated by previously published ACB scores. Potential reasons for those drugs missing could be that the drugs were mainly used in Germany and not internationally, e.g. Metamizol sodium, or that the drugs were new on the market and not previously analysed, e.g. Apixaban. To address missing and potentially new anticholinergic drugs, updates are planned in follow-up projects.

The validity of selection of drugs with anticholinergic activity and the grading can be questioned. Among the selected scores there is a great variety in study design and setting. Different methods to assess and rate anticholinergic activity were used: product information, specialised literature on ADRs, review of literature, expert opinion as well as serum radio receptor assay, dissociation constant for cholinergic receptor and other laboratory data [10, 18–24, 26, 40]. We worked with that variety by comparing different scores. As final decision on inclusion and rating of anticholinergic drugs was mainly a subjective decision of experts and not based on clinical outcomes, the ratings may be discussed further. Nevertheless, there is no approved methodology to measure the ACB and expert rating is preferred to measuring serum assays [27]. The list did not include topical, ophthalmic, otic or nasal drugs. It cannot be excluded that there might be systemic or local anticholinergic effects with these application routes.

The distinction of anticholinergic potency from 0 to 3 might not be the best method to quantify anticholinergic burden, but as most existing scores used this or a similar rating it was the only way to work with the existing lists [18, 19]. Through the upper limit of score 3, the suggested ceiling effect of the ACB was included [19]. This means that at a high ACB, drug effects are assumed to reach a plateau even when the number of anticholinergic drugs further increases [19]. For a more accurate evaluation of anticholinergic burden a finer distinction in some drug classes would be useful. Drug classes like tricyclic antidepressants or anticholinergics for urinary incontinence were all scored with a strong ACB (ACB = 3). Although those drugs all have a strong anticholinergic burden, some are more problematic than others. For example, Trospium should have less central reactions than other anticholinergics for urinary incontinence because of its quaternary chemical structure, but it still shows anticholinergic adverse effects and was rated having strong anticholinergic properties by Boustani et al. [40]*.* Another method to further refine the evaluation of ACB would be to consider the dosages of anticholinergic drugs via the Drug Burden Index [33]. A recently published cohort study of German older outpatients found a significant association of the drug Burden Index with Mini-Mental State Examination Score, Barthel index, Falls and use of laxatives [42]. We did not use this approach as it is more complicated and time-consuming to use in daily routine and it is not compatible with the scores used [11]. Our decision to apply the higher score (Fig. 2 step 3) might be questioned due to the automatic application of a higher anticholinergic rating. We decided to take this approach in order to avoid missing any drugs with anticholinergic properties.

Although a high anticholinergic burden should be avoided if possible, the deprescribing of anticholinergic drugs is not always possible. Some indications like for example urinary incontinence or some psychiatric indications require anticholinergic medications that cannot be easily subsidised by other non-anticholinergic drugs due to clinical reasons. While urinary incontinence can be handled well with non-pharmacological options, if pharmacological treatment is required anticholinergic drugs are the best options. Thus, this list should be considered as decision support for the prescriber rather than as a strict deprescribing directive.

## Conclusion

Although anticholinergic burden is only one factor of many to consider in multimorbid geriatric patients, it is important to discuss anticholinergic burden and its effects. This list can be used in Germany and countries with similar drugs approved to assess the anticholinergic burden of geriatric patients. It is valuable for prescribers to use in the daily clinical setting as only drugs available in Germany are listed and data from different studies is merged into one table so that a quick overview is possible. Further cluster-randomised studies investigating whether the implementation of the list reduces anticholinergic side effects, falls or delirium are necessary for its validation.

## Additional file

Additional file 1: **Table S1.** Drugs extracted from published tools and excluded because they are not available in Germany. NB: This table extends to four pages. **Table S2.** Drugs extracted from published tools and excluded because they are topical, nasal, ophthalmic, or otic drugs or drug combinations. NB: This table extends to two pages. **Table S3.** All drugs scored 0. NB: This table extends to eight pages. (DOCX 157 kb)

## Abbreviations

ACB: Anticholinergic burden
ADR: Adverse drug reaction
ATC: Anatomical, Therapeutic and Chemical
