# Supplementary material for: An anticholinergic burden score for German prescribers: score development
Source: BMC Geriatr. 2018 Oct 11;18:239. doi: 10.1186/s12877-018-0929-6 (PMC6180424; doi:10.1186/s12877-018-0929-6)
Supplement: Supplementary file 1 — Table S1. Drugs extracted from published tools and excluded because they are not available in Germany. NB: This table extends to four pages. Table S2. Drugs extracted from published tools and excluded because they are topical, nasal, ophthalmic, or otic drugs or drug combinations. NB: This table extends to two pages. Table S3. All drugs scored 0. NB: This table extends to eight pages. (DOCX 157 kb) [file 12877_2018_929_MOESM1_ESM.docx]

Table S1. Drugs extracted from published tools and excluded because they are not available in Germany. NB: This table extends to four pages.

| **Drug** | **ATC-Code** | **Carnahan 2006 USA [**[**1**](#_ENREF_1)**]** | **Ancelin 2006 France [**[**2**](#_ENREF_2)**]** | **Rudolph 2008 USA [**[**3**](#_ENREF_3)**]** | **Han 2008 USA [**[**4**](#_ENREF_4)**]** | **Ehrt 2010 Norway modified [**[**5**](#_ENREF_5)**]** | **Sittironnarit 2011 Australia [**[**6**](#_ENREF_6)**]** | **Boustani 2012 USA [**[**7**](#_ENREF_7)**]** | **Duran 2013 [**[**8**](#_ENREF_8)**]** |
| --- | --- | --- | --- | --- | --- | --- | --- | --- | --- |
| Acepromazine | N05AA04 |  | 3 |  |  |  |  |  | 3 |
| Aceprometazine | - |  | 3 |  |  |  |  |  | 3 |
| Acetaminophen/Dichloralphenazone/Isometheptene | - | 0 |  |  |  |  |  |  |  |
| Alimemazine | R06AD01 |  | 2 |  |  | 0 |  | 1 | 2 |
| Aluminum Carbonate | - | 0 |  |  |  |  |  |  |  |
| Alverine | A03AX08 |  | 2 |  |  |  |  | 1 | 0 or 1 |
| Ammonium Lactate Topical | - | 0 |  |  |  |  |  |  |  |
| Amoxapine | N06AA17 |  | 3 |  |  |  |  | 3 | discrepant |
| Anileridine | N01AH05 | 0 |  |  |  |  |  |  |  |
| Antioxidant | - |  |  |  |  |  | 0 |  |  |
| Bacitracin Ophthalmic | - | 0 |  |  |  |  |  |  |  |
| Bacitracin Topical | D06AX05 | 0 |  |  |  |  |  |  |  |
| Belladonna alkaloids | A03BA04 |  | 3 |  | 3 |  |  | 2 | 3 |
| Benefibre | - |  |  |  |  |  | 0 |  |  |
| Benzonatate | R05DB01 | 0 |  |  |  |  |  |  |  |
| Benztropine | N04AC01 | 3 |  | 3 |  | 3 |  | 3 | 3 |
| Bismuth Subsalicylate | A02BX22 | 0 |  |  |  |  |  |  |  |
| Brahmi | - |  |  |  |  |  | 0 |  |  |
| Brompheniramine | R06AB01 | 3 |  |  |  |  |  | 3 | 3 |
| Brompheniramine maleate | R06AB01 |  |  |  |  |  |  |  |  |
| Bumetanide | C03CA02 | 0 |  |  |  | 0 |  |  |  |
| Butabarbital; Secbutabarbital | - | 0 |  |  |  |  |  |  |  |
| Butalbital | - | 0 |  |  |  |  |  |  |  |
| Camphor-Menthol Topical | - | 0 |  |  |  |  |  |  |  |
| Carbachol Ophthalmic | S01EB02 | 0 |  |  |  |  |  |  |  |
| Carbamide Peroxide Otic | D02AE02 | 0 |  |  |  |  |  |  |  |
| Carbinoxamine | R06AA08 | 3 |  |  |  |  |  | 3 | 3 |
| Carisoprodol | M03BA02 | 0 |  | 3 |  |  |  |  | discrepant |
| Casanthranol | - | 0 |  |  |  |  |  |  |  |
| Casanthranol-Docusate | - | 0 |  |  |  |  |  |  |  |
| Cascara Sagrada | - | 0 |  |  |  |  |  |  |  |
| Cefamandole | J01DC03 | 1 |  |  |  |  |  |  | 0 or 1 |
| Cefoxitin | J01DC01 | 1 |  |  |  |  |  |  | 0 or 1 |
| Cephalothin | J01DB03 | 1 |  |  |  |  |  |  | 0 or 1 |
| Cerivastatin | C10AA06 | 0 |  |  |  |  |  |  |  |
| Chlorpromazine | N05AA01 | 3 |  | 3 | 3 |  |  | 3 | 3 |
| Chlorpropamide | A10BB02 | 0 |  |  |  |  |  |  |  |
| Chlorzoxazone | M03BB03 | 0 |  |  |  |  |  |  |  |
| Choline Salicylate | N02BA03 |  |  |  |  |  | 0 |  |  |
| Cisapride | A03FA02 | 0 |  |  |  |  |  |  |  |
| Clavulanate | - | 0 |  |  |  |  |  |  |  |
| Clidinium | A03CA02 |  |  |  |  |  |  | 1 |  |
| Colestipol | C10AC02 | 0 |  |  |  |  |  |  |  |
| Cortisone | H02AB10 | 1 |  |  |  |  |  |  | 0 or 1 |
| Cyclobenzaprine | M03BX08 | 2 |  | 2 | 1 |  |  | 2 | 2 |
| Cycloserine | J04AB01 | 1 |  |  |  |  |  |  | 0 or 1 |
| Danazol | G03XA01 | 0 |  |  |  |  | 0 |  |  |
| Dehydroepiandrosterone; Prasteron | A14AA07 |  |  |  |  |  | 0 |  |  |
| Demeclocycline | J01AA01 | 0 |  |  |  |  |  |  |  |
| Desipramine | N06AA01 | 3 |  | 2 | 2 |  |  | 3 | 3 |
| Dexchlorpheniramine | R06AB02 |  | 3 |  |  |  | 3 |  | 3 |
| Dicyclomine | A03AA07 | 3 |  | 3 |  |  |  | 3 | 3 |
| Dienestrol Topical | G03CB01 | 0 |  |  |  |  |  |  |  |
| Diflunisal | N02BA11 | 0 |  |  |  |  |  |  |  |
| Dihydroxyaluminum Sodium Carbonate | A02AF03 | 0 |  |  |  |  |  |  |  |
| Diphenoxylate | A07DA01 | 0 |  |  |  |  | 0 |  | 0 or 1 |
| Dipivefrin Ophthalmic | S01EA02 | 0 |  |  |  |  |  |  |  |
| Dirithromycin | J01FA13 | 0 |  |  |  |  |  |  |  |
| Disopyramide | C01BA03 | 2 |  |  |  | 0 | 0 | 1 | 2 |
| Disulfiram | N07BB01 |  |  |  |  |  | 0 |  |  |
| Dothiepin | N06AA16 |  |  |  |  |  | 2 |  | 2 |
| Edrophonium | - | 0 |  |  |  |  |  |  |  |
| Eformetalol Fumarate | - |  |  |  |  |  | 0 |  |  |
| Emepronium | G04BD01 |  |  |  |  | 3 |  |  | 3 |
| Ergoloid Mesylates | C04AE01 | 0 |  |  |  |  |  |  |  |
| Estazolam | N05CD04 | 1 |  |  |  |  |  |  | 0 or 1 |
| Esterified Estrogens | - | 0 |  |  |  |  |  |  |  |
| Estradiol Topical | - | 0 |  |  |  |  |  |  |  |
| Estropipate; Piperazine Estrone Sulfate | P02CB01 | 0 |  |  |  |  | 0 |  |  |
| Etodolac | M01AB08 | 0 |  |  |  |  |  |  |  |
| Fluorometholone | C05AA06 |  |  |  |  |  | 0 |  |  |
| Halcinonide Topical | D07AD02 | 0 |  |  |  |  |  |  |  |
| Herb | - |  |  |  |  |  | 0 |  |  |
| Homatropine | S01FA05 |  |  |  | 3 |  |  |  | 3 |
| Hormone Replacement Therapy | - |  |  |  |  |  | 0 |  |  |
| Hydrocodone | R05DA03 | 0 |  |  | 2 |  |  |  | 2 |
| Hydrocortisone Otic | - | 0 |  |  |  |  |  |  |  |
| Hydroxylpropyl Methylcellulose Ophthalmic | - | 0 |  |  |  |  |  |  |  |
| Hyoscyamine | A03BA03 | 3 |  | 3 |  |  |  | 3 | 3 |
| Iloperidone | N05AX14 |  |  |  |  |  |  | 1 |  |
| Intestinal Flora | - |  |  |  |  |  | 0 |  |  |
| Iron Polysaccharide | - | 0 |  |  |  |  |  |  |  |
| Isosorbide | - | 1 |  |  |  |  |  | 1 |  |
| Labetalol | C07AG01 | 0 |  |  |  |  | 0 |  |  |
| Lactic Acid Bacteria | A07FA01 |  |  |  |  |  | 0 |  |  |
| Lactobacillus Rhamnosus | - |  |  |  |  |  | 0 |  |  |
| Lindane Topical | P03AB02 | 0 |  |  |  |  |  |  |  |
| Lumiracoxib | M01AH06 |  |  |  |  |  | 1 |  | 0 or 1 |
| LVP Solution | H01BA03 | 0 |  |  |  |  |  |  |  |
| Meclizine/Meclozine | R06AE05 | 3 |  | 3 |  |  |  | 3 | 3 |
| Meprobamate | N05BC01 | 0 |  |  |  |  |  |  |  |
| Metaxalone | - | 0 |  |  |  |  |  |  |  |
| Methazolamide | A01EC05 | 0 |  |  |  |  |  |  |  |
| Methyclothiazide | C03AA08 | 0 |  |  |  |  |  |  |  |
| Methylcellulose | A06AC06 | 0 |  |  |  |  |  |  |  |
| Methyltestosterone | G03BA02 | 0 |  |  |  |  |  |  |  |
| Metolazone | C03BA08 | 0 |  |  |  |  |  |  |  |
| Mineral | - |  |  |  |  |  | 0 |  |  |
| Mineral Oil | - | 0 |  |  |  |  |  |  |  |
| Molindone | N05AE02 | 2 |  |  |  |  |  | 2 | 2 |
| Multiple Actives | - |  |  |  |  |  | 0 |  |  |
| Nadolol | C07AA12 | 0 |  |  |  |  |  |  |  |
| Naturopathic Agent | - |  |  |  |  |  | 0 |  |  |
| Nefazodone | N06AX06 | 0 |  |  | 1 |  |  |  | 2 |
| Nefopam | N02BG06 |  |  |  |  |  |  | 2 |  |
| Nizatidine | A02BA0discrepant | 1 |  |  |  |  | 0 | 0 | 0 or 1 |
| Pancrelipase | A09AA02 | 0 |  |  |  |  | 0 |  |  |
| Penicillin | - | 0 |  |  |  |  |  |  |  |
| Phenazopyridine | G04BX06 | 0 |  |  |  |  |  |  |  |
| Phenelzine | N06AF03 | 1 |  |  |  |  |  |  | 2 |
| Phenyl Salicylate | G04BX12 | 0 |  |  |  |  |  |  |  |
| Pirbuterol | R03AC08 | 0 |  |  |  |  |  |  |  |
| Pivampicillin | J01CA02 | 0 |  |  |  |  |  |  |  |
| Pizotifen | N02CX01 |  |  |  |  |  | 0 |  |  |
| Pramoxine Topical; Pramocaine | C05AD07 | 0 |  |  |  |  |  |  |  |
| Prazosin | C02CA01 | 0 |  |  |  |  | 0 |  |  |
| Procainamide | C01BA02 | 0 |  |  |  |  |  |  |  |
| Prochlorperazine | N05AB04 | 1 |  | 2 | 2 | 0 | 2 |  | 2 |
| Promazine | N05AA03 |  |  |  |  | 2 |  |  | 2 |
| Propantheline | A03AB05 | 3 |  |  | 2 |  |  | 3 | 3 |
| Propentheline | - |  |  |  |  |  |  |  |  |
| Propoxyphene | N02AC0discrepant | 0 |  |  | 2 | 1 |  |  | 2 |
| Protriptyline/Protyptyline | N06AA11 | 3 |  |  |  |  | 3 |  | 3 |
| Pyrilamine | R06AC01 | 3 |  |  |  |  |  |  | 3 |
| Rimantadine | J05AC02 | 0 |  |  |  |  |  |  |  |
| Rofecoxib | M01AH02 | 0 |  |  |  |  |  |  |  |
| Rosiglitazone | A10BG02 | 0 |  |  |  |  | 0 |  | 0 |
| Salsalate | N02BA06 | 0 |  |  |  |  |  |  |  |
| Sodium Citrotartrate | A06AD21 |  |  |  |  |  | 0 |  |  |
| Sterculia | A06AC03 |  |  |  |  |  | 0 |  |  |
| Sulfacetamide sodium | D10AF06 |  |  |  |  |  | 0 |  |  |
| Sulfamethizole | B05CA0discrepant | 0 |  |  |  |  |  |  |  |
| Sulindac | M01AB02 | 0 |  |  |  | 0 |  |  |  |
| Tacrine | N06DA01 | 0 |  |  |  |  |  |  |  |
| Terconazole Topical | G01AG02 | 0 |  |  |  |  |  |  |  |
| Thiothixene | N05AF04 | 1 |  | 3 |  |  |  |  | 3 |
| Thyroid Desiccated | H03AA05 | 0 |  |  |  |  |  |  |  |
| Thyroid Supplement | - |  |  |  |  |  | 0 |  |  |
| Trichlormethiazide | C03AA06 | 0 |  |  |  |  |  |  |  |
| Trifluoperazine | N05AB06 | 1 |  | 3 |  |  |  | 3 | discrepant |
| Troglitazone | A10BG01 | 0 |  |  |  |  |  |  |  |
| Tropatepine | N04AA12 |  | 3 |  |  |  |  |  | 3 |
| Vitamin | - |  |  |  |  |  | 0 |  |  |
| Zafirlukast | R03DC01 | 0 |  |  |  |  |  |  |  |

Table S2. Drugs extracted from published tools and excluded because they are topical, nasal, ophthalmic, or otic drugs or drug combinations. NB: This table extends to two pages.

| **Drug** | **ATC-Code** | **Carnahan 2006 USA [**[**1**](#_ENREF_1)**]** | **Ancelin 2006 France [**[**2**](#_ENREF_2)**]** | **Rudolph 2008 USA [**[**3**](#_ENREF_3)**]** | **Han 2008 USA [**[**4**](#_ENREF_4)**]** | **Ehrt 2010 Norway modified [**[**5**](#_ENREF_5)**]** | **Sittironnarit 2011 Australia [**[**6**](#_ENREF_6)**]** | **Boustani 2012 USA [**[**7**](#_ENREF_7)**]** | **Duran 2013 [**[**8**](#_ENREF_8)**]** |
| --- | --- | --- | --- | --- | --- | --- | --- | --- | --- |
| Acetic Acid Topical | G01AD02 | 0 |  |  |  |  |  |  |  |
| Apraclonidine Ophthalmic | S01EA03 | 0 |  |  |  |  |  |  |  |
| Balsam Peru Topical | - | 0 |  |  |  |  |  |  |  |
| Beclomethasone Nasal | R01AD01 | 0 |  |  |  |  |  |  |  |
| Benzocaine Topical | C05AD03 | 0 |  |  |  |  |  |  |  |
| Betamethasone Topical | C05AA05 | 0 |  |  |  |  |  |  |  |
| Betamethasone-clotrimazole Topical | - | 0 |  |  |  |  |  |  |  |
| Betaxolol Ophthalmic | S01ED02 | 0 |  |  |  |  |  |  |  |
| Brimonidine Ophthalmic | S01EA05 | 0 |  |  |  |  |  |  |  |
| Brinzolamide Ophthalmic | S01EC04 | 0 |  |  |  |  | 0 |  |  |
| Budesonide Nasal | R01AD05 | 0 |  |  |  |  |  |  |  |
| Calamine Topical; Zinc Oxide | - | 0 |  |  |  |  |  |  |  |
| Calcipotriene Topical; Calcipotriol | D05AX02 | 0 |  |  |  |  |  |  |  |
| Calcium and Viatmin D | A12AX | 0 |  |  |  |  |  |  |  |
| Carbidopa-Levodopa | N04BA02 | 0 |  | 1 | 1 |  | 1 |  | 0 or 1 |
| Cetylpyridinium Topical | B05CA01 | 0 |  |  |  |  |  |  |  |
| Chlorhexidine Topical | A01AB03 | 0 |  |  |  |  |  |  |  |
| Ciclopirox Topical | D01AE14 | 0 |  |  |  |  |  |  |  |
| Clindamycin Topical | D10AF01 | 0 |  |  |  |  |  |  |  |
| Collagenase Topical | D03BA02 | 0 |  |  |  |  |  |  |  |
| Desonide Topical | D07AB08 | 0 |  |  |  |  |  |  |  |
| Desoximetasone Topical | D07AC03 | 0 |  |  |  |  |  |  |  |
| Dexamethasone Nasal | R01AD03 | 0 |  |  |  |  |  |  |  |
| Dexamethasone Ophthalmic | S01BA01 | 0 |  |  |  |  |  |  |  |
| Dexamethasone Topical | - | 0 |  |  |  |  |  |  |  |
| Dorzolamide Ophthalmic | S01EC03 | 0 |  |  |  |  | 0 |  |  |
| Econazole Topical | D01AC03 | 0 |  |  |  |  |  |  |  |
| Empracet (Acetaminophen +Codeine Phosphate) | N02AJ06 |  |  |  |  |  |  | 2 |  |
| Fluocinonide Topical | C05AA11 | 0 |  |  |  |  |  |  |  |
| Fluoride Topical | A12CD01 | 0 |  |  |  |  |  |  |  |
| Fluorometholone Ophthalmic | S01CB05 | 0 |  |  |  |  |  |  |  |
| Fluticasone-salmeterol | R03AK06 | 1 |  |  |  |  |  |  | 0 or 1 |
| Gentamicin Ophthalmic | S03AA06 | 0 |  |  |  |  |  |  |  |
| Gentamicin Topical | D06AX07 | 0 |  |  |  |  |  |  |  |
| Glycerin Topical | A06AG04 | 0 |  |  |  |  |  |  |  |
| Hydrocortisone Ophthalmic | S01BA02 | 0 |  |  |  |  |  |  |  |
| Hydrocortisone Topical | C05AA01 | 0 |  |  |  |  |  |  |  |
| Ketoconazole Topical | D01AC08 | 0 |  |  |  |  |  |  |  |
| Ketotifen Ophthalmic | S01GX08 | 1 |  |  |  |  |  |  | 0 or 1 |
| Lanolin-Mineral Oil Topical | - | 0 |  |  |  |  |  |  |  |
| Latanoprost Ophthalmic | S01EE01 | 0 |  |  |  |  | 0 |  |  |
| Levobunolol Ophthalmic | S01ED03 | 0 |  |  |  |  |  |  |  |
| Loteprednol Ophthalmic | S01BA14 | 0 |  |  |  |  |  |  |  |
| Methylprednisolone Topical | D10AA02 | 0 |  |  |  |  |  |  |  |
| Miconazole Topical | D01AC02 | 0 |  |  |  |  |  |  |  |
| Mometasone Nasal | R01AD09 | 0 |  |  |  |  |  |  |  |
| Mupirocin Topical | D06AX09 | 0 |  |  |  |  |  |  |  |
| Neomycin Ophthalmic | S03AA01 | 0 |  |  |  |  |  |  |  |
| Olopatadine Ophthalmic | S01GX09 | 0 |  |  |  |  |  |  |  |
| Oxymetazoline Nasal | S01GA04 | 0 |  |  |  |  |  |  |  |
| Permethrin Topical | P03AC04 | 0 |  |  |  |  |  |  |  |
| Petrolatum Topical | D02AC | 0 |  |  |  |  |  |  |  |
| Pilocarpine Ophthalmic | S01EB01 | 0 |  |  |  |  |  |  |  |
| Polymyxin B Ophthalmic | S01AA18 | 0 |  |  |  |  |  |  |  |
| Prednisolone Ophthalmic | S01BA04 | 0 |  |  |  |  |  |  |  |
| Pseudoephedrine-Triprolidine | R01BA52 |  |  | 2 |  |  |  |  |  |
| Rimexolone Ophthalmic | S01BA13 | 0 |  |  |  |  |  |  |  |
| Salicylic Acid Topical | D01AE12 | 0 |  |  |  |  |  |  |  |
| Selenium Sulfide Topical | D01AE13 | 0 |  |  |  |  |  |  |  |
| Silver Sulfadiazine Topical | D06BA01 | 0 |  |  |  |  |  |  |  |
| Sodium Sulfacetamide Ophthalmic | S01AB04 | 0 |  |  |  |  | 0 |  |  |
| Terbinafine Topical | D01AE15 | 0 |  |  |  |  |  |  |  |
| Tobramycin Ophthalmic | S01AA12 | 0 |  |  |  |  |  |  |  |
| Triamcinolone Nasal | R01AD11 | 0 |  |  |  |  |  |  |  |
| Triamcinolone Topical | C05AA12 | 0 |  |  |  |  |  |  |  |
| Triethanolamine Polypeptide Oleate Otic | - | 0 |  |  |  |  |  |  |  |

Table S3. All drugs scored 0. NB: This table extends to eight pages.

| **Drug** | **ATC-Code** | **Carnahan 2006 USA [**[**1**](#_ENREF_1)**]** | **Ancelin 2006 France [**[**2**](#_ENREF_2)**]** | **Rudolph 2008 USA [**[**3**](#_ENREF_3)**]** | **Han 2008 USA [**[**4**](#_ENREF_4)**]** | **Ehrt 2010 Norway modified [**[**5**](#_ENREF_5)**]** | **Sittironnarit 2011 Australia [**[**6**](#_ENREF_6)**]** | **Boustani 2012 USA [**[**7**](#_ENREF_7)**]** | **Duran 2013 [**[**8**](#_ENREF_8)**]** |
| --- | --- | --- | --- | --- | --- | --- | --- | --- | --- |
| Acarbose | A10BF01 | 0 |  |  |  |  |  |  |  |
| Acetazolamide | S01EC01 | 0 |  |  |  |  |  |  |  |
| N-Acetyl-L-Cysteine | R05CB01 |  |  |  |  |  | 0 |  |  |
| Acetylsalicylic Acid; Aspirin | N02BA01 | 0 |  |  |  | 0 | 0 |  | 0 |
| Acyclovir | S01AD03 | 0 |  |  |  |  |  |  |  |
| Acitratin | D05BB02 |  |  |  |  |  | 0 |  |  |
| Adenosine | C01EB10 | 0 |  |  |  |  |  |  |  |
| Agomelatine | N06AX22 |  |  |  |  |  |  |  |  |
| Alendronate | M05BA04 | 0 |  |  |  |  |  |  |  |
| Allopurinol | M04AA01 | 0 |  |  |  | 0 | 0 |  | 0 |
| Aluminum Hydroxide | V03AE12 | 0 |  |  |  |  | 0 |  |  |
| Amiloride | C03DB01 | 0 |  |  |  |  | 0 |  |  |
| Amiodarone | C01BD01 | 0 |  |  |  |  | 0 |  |  |
| Amlodipine | C08CA01 | 0 |  |  |  | 0 | 0 |  | 0 |
| Amoxicillin | J01CA04 | 0 |  |  |  |  | 0 |  | 0 or 1 |
| Amoxicillin-Clavulanate | J01CR02 | 0 |  |  |  |  |  |  |  |
| Anagrelide | L01XX35 | 0 |  |  |  |  |  |  |  |
| Anastrozole | L02BG03 | 0 |  |  |  |  | 0 |  |  |
| Apixaban | B01FAF02 |  |  |  |  |  |  |  |  |
| Ascorbic Acid | G01AD03 | 0 |  |  |  |  |  |  |  |
| Atorvastatin | C10AA05 | 0 |  |  |  | 0 | 0 |  | 0 |
| Azithromycin | J01FA10 | 0 |  |  |  |  |  |  |  |
| Beclomethasone | A07EA07 | 0 |  |  |  |  | 0 |  |  |
| Beta-Carotene | D02BB01 | 0 |  |  |  |  |  |  |  |
| Betahistine | N07CA01 |  |  |  |  |  | 0 |  |  |
| Betaine | A16AA06 |  |  |  |  |  | 0 |  |  |
| Betamethasone Dipropionat | C05AA05 |  |  |  |  |  | 0 |  |  |
| Betamethasone Valerate | C05AA05 |  |  |  |  |  | 0 |  |  |
| Bethanechol | N07AB02 | 0 |  |  |  |  |  |  |  |
| Bicalutamide | L02BB03 | 0 |  |  |  |  |  |  |  |
| Bimatoprost | S01EE03 |  |  |  |  |  | 0 |  |  |
| Bisoprolol | C07AB07 | 0 |  |  |  |  | 0 |  |  |
| Brimonidine | D11AX21 |  |  |  |  |  | 0 |  |  |
| Bromhexine | R05CB02 |  |  |  |  |  | 0 |  |  |
| Budesonide | A07EA06 | 0 |  |  |  |  | 0 |  |  |
| Buprenorphine | N02AE01 |  |  |  |  | 0 | 0 |  |  |
| Buspirone | N05BE01 | 0 |  |  |  |  |  |  |  |
| Cabergoline | G02CB03 |  |  |  |  | 0 |  |  |  |
| Calcitonin | H05BA01 | 0 |  |  |  |  |  |  |  |
| Calcitriol | A11CC04 | 0 |  |  |  |  |  |  |  |
| Calcium Acetate | V03AE07 | 0 |  |  |  |  |  |  |  |
| Calcium Carbonate | A02AC01 | 0 |  |  |  |  |  |  |  |
| Candesartan | C09CA06 | 0 |  |  |  |  | 0 |  |  |
| Cilexetil | - |  |  |  |  |  | 0 |  |  |
| Carbimazole | H03BB01 |  |  |  |  |  | 0 |  |  |
| Carmellose Sodium | S01XA20 |  |  |  |  |  | 0 |  |  |
| Carnitine | A16AA01 |  |  |  |  |  | 0 |  |  |
| Carvedilol | C07AG02 | 0 |  |  |  |  | 0 |  |  |
| Cefaclor | J01DC04 | 0 |  |  |  |  |  |  |  |
| Cephalexin | J01DB01 | 0 |  |  |  |  | 0 |  | 0 or 1 |
| Cefazolin | J01DB04 | 0 |  |  |  |  |  |  |  |
| Ceftibuten | J01DD14 | 0 |  |  |  |  |  |  |  |
| Cefixime | J01DD08 | 0 |  |  |  |  |  |  |  |
| Ceftriaxone | J01DD04 | 0 |  |  |  |  |  |  |  |
| Cefuroxime | J01DC02 | 0 |  |  |  |  |  |  |  |
| Quinine | P01BC01 | 0 |  |  |  |  | 0 |  |  |
| Chloral Hydrate | N05CC01 | 0 |  |  |  |  | 0 |  |  |
| Chlorambucil | L01AA02 | 0 |  |  |  | 0 |  |  |  |
| Chloramphenicol | D06AX02 |  |  |  |  |  | 0 |  |  |
| Chlorothiazide | C03AA04 | 0 |  |  |  |  |  |  |  |
| Chlorprotixen | N05AF03 |  |  |  |  | 0 |  |  |  |
| Chondroitin | M01AX25 | 0 |  |  |  |  |  |  |  |
| Cilostazol | B01AC23 | 0 |  |  |  |  |  |  |  |
| Ciprofloxacin | S03AA07 | 0 |  |  |  |  |  |  |  |
| Clarithromycin | J01FA09 | 0 |  |  |  |  |  |  |  |
| Clobazam | N05BA09 | 0 |  |  |  |  |  |  |  |
| Clodronate | M05BA02 | 0 |  |  |  |  |  |  |  |
| Clomethiazole | N05CM02 |  |  |  |  | 0 |  |  |  |
| Clonidine | S01EA04 | 0 |  |  |  |  | 0 |  |  |
| Clopidogrel | B01AC04 | 0 |  |  |  |  | 0 |  | 0 |
| Clotrimazole | G01AF02 | 0 |  |  |  |  |  |  |  |
| Cloxacillin | J01CF02 | 0 |  |  |  |  |  |  |  |
| Caffeine | N06BC01 | 0 |  |  |  |  |  |  |  |
| Colchicine | M04AC01 | 0 | 3 |  |  |  | 0 | 1 | discrepant |
| Colecalciferol | A11CC05 |  |  |  |  |  |  |  |  |
| Cholestyramine | C10AC01 | 0 |  |  |  |  |  |  |  |
| Cromolyn; Sodium Chromoglycate | A07EB01 | 0 |  |  |  |  | 0 |  |  |
| Cyanocobalamin | B03BA01 | 0 |  |  |  |  |  |  |  |
| Cyclophosphamide | L01AA01 | 0 |  |  |  |  |  |  |  |
| Dabigatran | B01AE07 |  |  |  |  |  |  |  |  |
| Dantrolene | M03CA01 | 0 |  |  |  |  |  |  |  |
| Darbepoetin | B03XA02 |  |  |  |  |  |  |  |  |
| Deferasirox | V03AC03 |  |  |  |  |  | 0 |  |  |
| Desmopressin | H01BA02 | 0 |  |  |  |  |  |  |  |
| Dextran 70 | B05AA05 |  |  |  |  |  | 0 |  |  |
| Dextroproproxyphene | N02AC04 |  |  |  |  |  | 0 |  |  |
| Diclofenac | D11AX18 | 0 |  |  |  |  | 0 |  |  |
| Dimeticone | P03AX05 |  |  |  |  | 0 |  |  |  |
| Dobutamine | C01CA07 | 0 |  |  |  |  |  |  |  |
| Docusate Sodium; Docusate | A06AA02 | 0 |  |  |  |  | 0 |  |  |
| Donepezil | N06DA02 | 0 |  |  |  | 0 | 0 |  | 0 |
| Dopamine | C01CA04 | 0 |  |  |  |  |  |  |  |
| Doxazosin | C02CA04 | 0 |  |  |  |  |  |  |  |
| Doxycycline | A01AB22 | 0 |  |  |  |  | 0 |  |  |
| Dulaglutide | A10BJ05 |  |  |  |  |  |  |  |  |
| Duloxetine | N06AX21 | 0 |  |  |  |  | 0 | 0 | 0 or 1 |
| Dydrogesterone | G03DB01 |  |  |  |  |  | 0 |  |  |
| Edoxaban | B01AF03 |  |  |  |  |  |  |  |  |
| Ferrous Gluconate | B03AA03 | 0 |  |  |  |  |  |  |  |
| Ferrous Sulfate | B03AA07 | 0 |  |  |  |  |  |  |  |
| Empagliflozin | A10BK03 |  |  |  |  |  |  |  |  |
| Enalapril | C09AA02 | 0 |  |  |  | 0 | 0 |  | 0 |
| Enoxaparin | B01AB05 | 0 |  |  |  |  | 0 |  |  |
| Adrenaline | A01AD01 |  |  |  |  |  | 0 |  |  |
| Eprosartan | C09CA02 |  |  |  |  |  | 0 |  |  |
| Ergocalciferol | A11CC01 | 0 |  |  |  |  |  |  |  |
| Erythromycin | D10AF02 | 0 |  |  |  |  | 0 |  |  |
| Epoetin Alfa | B03XA01 | 0 |  |  |  |  |  |  |  |
| Esomeprazole | A02BC05 | 0 |  |  |  |  | 0 |  |  |
| Estradiol; Oestradiol | G03CA03 | 0 |  |  |  |  | 0 |  |  |
| Estriol | G03CA04 |  |  |  |  | 0 | 0 |  |  |
| Ethambutol | J04AK02 | 0 |  |  |  |  |  |  |  |
| Ethinyl Estradiol | G03CA01 | 0 |  |  |  |  |  |  |  |
| Etidronate | M05BA01 | 0 |  |  |  |  |  |  |  |
| Ezetimibe | C10AX09 |  |  |  |  |  | 0 |  |  |
| Famciclovir | J05AB09 |  |  |  |  |  | 0 |  |  |
| Frangula | V04CZ05 |  |  |  |  |  | 0 |  |  |
| Felbamate | N03AX10 | 0 |  |  |  |  |  |  |  |
| Felodipine | C08CA02 | 0 |  |  |  |  | 0 |  |  |
| Fenofibrate | C10AB05 | 0 |  |  |  |  | 0 |  |  |
| Fenoterol inhalative | R03AC04 |  |  |  |  |  |  |  |  |
| Fentanyl Patch | - |  |  |  |  |  | 0 |  |  |
| Filgrastim | L03AA02 | 0 |  |  |  |  |  |  |  |
| Finasteride | D11AX10 | 0 |  |  |  |  | 0 |  |  |
| Fish Oil | - |  |  |  |  |  | 0 |  |  |
| Flecainide | C01BC04 | 0 |  |  |  |  | 0 |  |  |
| Flucloxacillin | J01CF05 |  |  |  |  |  | 0 |  |  |
| Fluconazole | D01AC15 | 0 |  |  |  |  |  |  |  |
| Fludrocortisone | H02AA02 | 0 |  |  |  |  |  |  |  |
| Flumazenil | V03AB25 | 0 |  |  |  |  |  |  |  |
| Flunisolide | R01AD04 | 0 |  |  |  |  |  |  |  |
| Fluorouracil | L01BC02 |  |  |  |  |  | 0 |  |  |
| Flutamide | L02BB01 | 0 |  |  |  |  |  |  |  |
| Fluticasone | D07AC17 | 0 |  |  |  |  | 0 |  |  |
| Fluvastatin | C10AA04 | 0 |  |  |  |  | 0 |  |  |
| Folic Acid | B03BB01 | 0 |  |  |  |  |  |  |  |
| Ginkgo | - | 0 |  |  |  |  | 0 |  |  |
| Formoterol inhalative | R03AC13 |  |  |  |  |  |  |  |  |
| Fosinopril | C09AA09 | 0 |  |  |  |  | 0 |  |  |
| Framycetin sulfate | D09AA01 |  |  |  |  |  | 0 |  |  |
| Gabapentin | N03AX12 | 0 |  |  |  |  | 0 | 0 |  |
| Galantamine | N06DA04 | 0 |  |  |  | 0 | 0 |  | 0 |
| Gemfibrozil | C10AB04 | 0 |  |  |  |  | 0 |  | 0 |
| Ginkgo Biloba | N06DP01 |  |  |  |  |  | 0 |  |  |
| Ginseng | A13AP02 |  |  |  |  |  | 0 |  |  |
| Glibenclamide; Glyburide | A10BB01 | 0 |  |  |  | 0 | 0 |  |  |
| Gliclazide | A10BB09 |  |  |  |  |  | 0 |  |  |
| Glimepiride | A10BB12 | 0 |  |  |  |  | 0 |  |  |
| Glipizide | A10BB07 | 0 |  |  |  | 0 | 0 |  | 0 |
| Glucagon | H04AA01 | 0 |  |  |  |  |  |  |  |
| Glucosamine | M01AX05 | 0 |  |  |  |  | 0 |  |  |
| Glucose | B05CX01 |  |  |  |  |  | 0 |  |  |
| Nitroglycerin, Glyceryl Trinitrate | C01DA02 | 0 |  |  |  | 0 | 0 |  | 0 |
| Goserelin Acetate | L02AE03 |  |  |  |  |  | 0 |  |  |
| Gramicidine | R02AB30 |  |  |  |  |  | 0 |  |  |
| Guanfacine | C02AC02 | 0 |  |  |  |  |  |  |  |
| Urea |  |  |  |  |  |  | 0 |  |  |
| Heparin | B01AB01 | 0 |  |  |  |  |  |  |  |
| Histidine | - |  |  |  |  |  | 0 |  |  |
| Hydrochlorothiazide | C03AA03 | 0 |  |  |  | 0 | 0 |  | 0 |
| Hydrocortisone Acetate | D07AA02 |  |  |  |  |  | 0 |  |  |
| Hydromorphone | N02AA03 | 0 |  |  |  |  |  |  |  |
| Hydroxyurea | L01XX05 | 0 |  |  |  |  |  |  |  |
| Hydroxychloroquin | P01BA02 | 0 |  |  |  |  | 0 |  |  |
| Hydroxypropyl Guar | S01XA20 |  |  |  |  |  | 0 |  |  |
| Hypromellose | S01KA02 |  |  |  |  |  | 0 |  |  |
| Ibuprofen | M01AE01 | 0 |  |  |  | 0 | 0 |  | 0 |
| Imipenem-Cilastatin | J01DH51 | 0 |  |  |  |  |  |  |  |
| Indapamide | C03BA11 | 0 |  |  |  |  | 0 |  |  |
| Indomethacin | M01AB01 | 0 |  |  |  |  | 0 |  |  |
| Influenza Virus Vaccine | J07BB |  |  |  |  |  | 0 |  |  |
| Inositol | A11HA07 |  |  |  |  |  | 0 |  |  |
| Insulin | A10A | 0 |  |  |  | 0 | 0 |  | 0 |
| Irbesartan | C09CA04 | 0 |  |  |  |  | 0 |  |  |
| Isoniazid | J04AC01 | 0 |  |  |  |  |  |  |  |
| Metamucil | A06AC01 |  |  |  |  |  | 0 |  |  |
| Psyllium | A06AC01 | 0 |  |  |  |  |  |  |  |
| Isradipine | C08CA03 | 0 |  |  |  |  |  |  |  |
| Potassium Chloride | A12BA01 | 0 |  |  |  |  |  |  |  |
| Potassium Citrate | A12BA02 | 0 |  |  |  |  |  |  |  |
| Potassium Bicarbonate | A12BA04 | 0 |  |  |  |  |  |  |  |
| Ketoprofen | M01AE03 | 0 |  |  |  | 0 | 0 |  | 0 |
| Colostrums | - |  |  |  |  |  | 0 |  |  |
| Conjugated Estrogens | G03CA57 | 0 |  |  |  |  | 0 |  |  |
| Lactulose | A06AD11 | 0 |  |  |  |  | 0 |  |  |
| Lamotrigine | N03AX09 | 0 |  |  |  |  |  |  |  |
| Lercanidipine | C08CA13 |  |  |  |  |  | 0 |  |  |
| Leuprolide | L02AE02 | 0 |  |  |  |  |  |  |  |
| Levetiracetam | N03AX14 |  |  |  |  |  |  | 0 |  |
| Levocabastine | R01AC02 |  |  |  |  |  | 0 |  |  |
| Levofloxacin | J01MA12 | 0 |  |  |  |  |  |  | 0 or 1 |
| Levothyroxine; Thyroxin; Thyroxin Sodium | H03AA01 | 0 |  |  |  | 0 | 0 |  |  |
| Lidocaine | C01BB01 | 0 |  |  |  |  |  |  |  |
| Liothyronine | H03AA02 | 0 |  |  |  |  |  |  |  |
| Lisinopril | C09AA03 | 0 |  |  |  | 0 | 0 |  | 0 |
| Losartan | C09CA01 | 0 |  |  |  | 0 |  |  | 0 |
| Lovastatin | C10AA02 | 0 |  |  |  |  |  |  |  |
| Lysine | B05XB03 | 0 |  |  |  |  |  |  |  |
| Polyethylene Glycol Electrolyte Solution | A06AD65 | 0 |  |  |  |  |  |  |  |
| Magnesium Preparations | A12CC30 | 0 |  |  |  |  |  |  |  |
| Mannitol | A06AD16 | 0 |  |  |  |  |  |  |  |
| Mebeverine | A03AA04 |  |  |  |  |  | 0 |  |  |
| Activated Charcoal | A07BA01 |  |  |  |  |  | 0 |  |  |
| Medroxyprogesterone | G03AC06 | 0 |  |  |  |  | 0 |  |  |
| Medroxyprogesterone Acetate | G03DA02 |  |  |  |  |  | 0 |  |  |
| Megestrol | G03AC05 | 0 |  |  |  |  |  |  |  |
| Melatonin | N05CH01 |  |  |  |  |  | 0 |  |  |
| Meloxicam | M01AC06 |  |  |  |  |  | 0 |  |  |
| Melperone | N05AD03 |  |  |  |  | 0 |  |  |  |
| Memantine | N06DX01 |  |  |  |  |  | 0 |  |  |
| Mesalazine; Mesalamine | A07EC02 | 0 |  |  |  |  | 0 |  |  |
| Metamizole | N02BB02 |  |  |  |  |  |  |  |  |
| Methenamine | J01XX05 | 0 |  |  |  |  |  |  |  |
| Methyldopa | C02AB | 0 |  |  |  |  |  |  |  |
| Methylene Blue | V03AB17 | 0 |  |  |  |  |  |  |  |
| Methylphenidate | N06BA04 | 0 |  |  |  |  |  |  |  |
| Metronidazole | A01AB17 | 0 |  |  |  |  | 0 |  |  |
| Mianserin | N06AX03 |  |  |  |  | 0 |  |  |  |
| Midodrine | C01CA17 | 0 |  |  |  |  |  |  |  |
| Minocycline | A01AB23 | 0 |  |  |  |  |  |  |  |
| Misoprostol | A02BB01 | 0 |  |  |  |  |  |  |  |
| Moclobemid | N06AG02 |  |  |  |  | 0 | 0 |  |  |
| Modafinil | N06BA07 |  |  |  |  |  | 0 |  |  |
| Nitroglycerin Transdermal Patch | C01DA02 |  |  |  |  |  | 0 |  |  |
| Moexipril | C09AA13 | 0 |  |  |  |  |  |  |  |
| Mometasone Furoate | D07AC13 |  |  |  |  |  | 0 |  |  |
| Montelukast | R03DC03 | 0 |  |  |  |  |  |  |  |
| Cranberry | - | 0 |  |  |  |  |  |  |  |
| Moxifloxacin | J01MA14 | 0 |  |  |  |  |  |  |  |
| Moxonidine | C02AC05 |  |  |  |  |  | 0 |  |  |
| Multivitamin | A11BA01 | 0 |  |  |  |  | 0 |  |  |
| Nabumetone | M01AX01 | 0 |  |  |  |  |  |  |  |
| Naloxone | V03AB15 | 0 |  |  |  |  |  |  |  |
| Naproxen | G02CC02 | 0 |  |  |  | 0 | 0 |  |  |
| Nateglinide | A10BX03 | 0 |  |  |  |  |  |  |  |
| Sodium Bicarbonate | B05CB04 | 0 |  |  |  |  |  |  |  |
| Sodium Chloride | B05CB01 | 0 |  |  |  |  |  |  |  |
| Sodium Phosphate | A06AD17 | 0 |  |  |  |  |  |  |  |
| Niacin | - | 0 |  |  |  |  |  |  |  |
| Nisoldipine | C08CA07 | 0 |  |  |  |  |  |  |  |
| Nitrazepam | N05CD02 |  |  |  |  | 0 | 0 |  |  |
| Nitrofurantoin | J01XE01 | 0 |  |  |  |  |  |  |  |
| Norepinephrine | C01CA03 | 0 |  |  |  |  |  |  |  |
| Norethisterone | G03AC01 |  |  |  |  |  | 0 |  |  |
| Norfloxacin | J01MA06 | 0 |  |  |  |  | 0 |  |  |
| Nystatin | A07AA02 | 0 |  |  |  |  |  |  |  |
| Octreotide | H01CB02 | 0 |  |  |  |  |  |  |  |
| Ofloxacin | J01MA01 | 0 |  |  |  |  |  |  |  |
| Olmesartan Medoxomil | C09CA08 |  |  |  |  |  | 0 |  |  |
| Omega-3 | C10AX06 |  |  |  |  |  | 0 |  |  |
| Omeprazole | A02BC01 | 0 |  |  |  | 0 | 0 |  | 0 |
| Pamidronate | M05BA03 | 0 |  |  |  |  |  |  |  |
| Pantoprazole | A02BC02 | 0 |  |  |  |  | 0 |  | 0 |
| Papaverine | A03AD01 | 0 |  |  |  |  |  |  |  |
| Paracetamol; Acetaminophen | N02BE01 | 0 |  |  |  | 0 | 0 |  | 0 |
| Pentazocine | N02AD01 |  |  |  |  | 0 |  |  |  |
| Pentoxifylline | C04AD03 | 0 |  |  |  |  |  |  |  |
| Pergolide | N04BC02 | 0 |  |  |  | 0 |  |  |  |
| Perindopril | C09AA04 | 0 |  |  |  |  | 0 |  |  |
| Phenprocoumon | B01AA04 |  |  |  |  |  |  |  |  |
| Phenylephrine | C01CA06 | 0 |  |  |  |  |  |  |  |
| Phenylpropanolamine | R01BA01 | 0 |  |  |  |  |  |  |  |
| Phenytoin | N03AB02 | 0 |  |  |  |  |  |  | 0 or 1 |
| Phytonadione; Vitamin K; Phylloquinone | B02BA01 | 0 |  |  |  |  |  |  |  |
| Pilocarpine | N07AX01 |  |  |  |  |  | 0 |  |  |
| Pindolol | C07AA03 | 0 |  |  |  |  |  |  |  |
| Pioglitazone | A10BG03 | 0 |  |  |  |  | 0 |  | 0 |
| Pipamperone | N05AD05 |  |  |  |  |  |  |  |  |
| Piritramide | N02AC03 |  |  |  |  |  |  |  |  |
| Piroxicam | M01AC01 | 0 |  |  |  | 0 | 0 |  | 0 |
| Polycarbophil | A06AC08 | 0 |  |  |  |  |  |  |  |
| Polyethelene Glycol | A06AD15 |  |  |  |  |  | 0 |  |  |
| Polyvinyl Alcohol | S01XC01 |  |  |  |  |  | 0 |  |  |
| Pravastatin | C10AA03 | 0 |  |  |  |  | 0 |  |  |
| Primidone | N03AA03 | 0 |  |  |  |  |  |  |  |
| Probenecid | M04AB01 | 0 |  |  |  |  | 0 |  |  |
| Progesterone | G03DA04 | 0 |  |  |  |  | 0 |  |  |
| Propafenone | C01BC03 | 0 |  |  |  |  |  |  |  |
| Propranolol | C07AA05 | 0 |  |  |  |  | 0 |  | 0 |
| Propylene Glycol | - |  |  |  |  |  | 0 |  |  |
| Propylthiouracil | H03BA02 | 0 |  |  |  |  |  |  |  |
| Protamine | V03AB14 |  |  |  |  |  | 0 |  |  |
| Pyrazinamide | J04AK01 | 0 |  |  |  |  |  |  |  |
| Pyridostigmine | N07AA02 | 0 |  |  |  |  |  |  |  |
| Pyridoxine | A11HA02 |  |  |  |  |  | 0 |  |  |
| Quinapril | C09AA06 | 0 |  |  |  |  | 0 |  |  |
| Rabeprazole | A02BC04 | 0 |  |  |  |  | 0 |  | 0 |
| Raloxifene | G03XC01 | 0 |  |  |  |  |  |  |  |
| Ramipril | C09AA05 | 0 |  |  |  |  | 0 |  |  |
| Reboxetine | N06AX18 |  |  |  |  |  | 0 |  |  |
| Repaglinide | A10BX02 | 0 |  |  |  |  |  |  |  |
| Reserpine | C02AA02 | 0 |  |  |  |  |  |  |  |
| Rifampin | J04AB02 | 0 |  |  |  |  |  |  |  |
| Risedronate | M05BA07 | 0 |  |  |  |  | 0 |  |  |
| Rivaroxaban | B01AF01 |  |  |  |  |  |  |  |  |
| Rivastigmine | N06DA03 |  |  |  |  |  | 0 |  |  |
| Castor Oil | A06AB05 | 0 |  |  |  |  |  |  |  |
| Ropinirole | N04BC04 | 0 |  |  |  | 0 | 0 |  | 0 |
| Rosuvastatin | C10AA07 |  |  |  |  |  | 0 |  |  |
| Roxithromycin | J01FA06 |  |  |  |  |  | 0 |  |  |
| Hydroxyethyrutosides | C05CA01 |  |  |  |  |  | 0 |  |  |
| Saccharomyces boulardii | A07FA02 |  |  |  |  |  |  |  |  |
| Salbutamol; Albuterol | R03AC02 | 0 |  |  |  | 0 | 0 |  |  |
| Salmeterol | R03AC12 | 0 |  |  |  |  | 0 |  |  |
| Senna (leaf); Sennosides A & B; Senokot | A06AB06 | 0 |  |  |  |  | 0 |  | 0 |
| Sevelamer | V03AE02 |  |  |  |  |  |  |  |  |
| Sildenafil Citrate | G04BE03 |  |  |  |  |  | 0 |  |  |
| Simethicone | A03AX13 | 0 |  |  |  |  | 0 |  |  |
| Simvastatin | C10AA01 | 0 |  |  |  | 0 | 0 |  | 0 |
| Sitagliptin | A10BH01 |  |  |  |  |  |  |  |  |
| Sotalol | C07AA07 | 0 |  |  |  |  | 0 |  |  |
| Spironolactone | C03DA01 | 0 |  |  |  | 0 | 0 |  | 0 |
| Sucralfate | A02BX02 | 0 |  |  |  |  |  |  |  |
| Sulfamethoxazole | J01EC01 | 0 |  |  |  |  |  |  |  |
| Sulfasalazine | A07EC01 |  |  |  |  |  | 0 |  |  |
| Succinylcholine | M03AB01 | 0 |  |  |  |  |  |  |  |
| Tadalafil | G04BE08 |  |  |  |  |  | 0 |  |  |
| Tamoxifen | L02BA01 | 0 |  |  |  | 0 | 0 | 0 | 0 |
| Tamsulosin | G04CA02 | 0 |  |  |  |  | 0 |  |  |
| Telmisartan | C09CA07 |  |  |  |  |  | 0 |  |  |
| Terazosin | G04CA03 | 0 |  |  |  |  |  |  |  |
| Terbinafine | D01BA02 |  |  |  |  |  | 0 |  |  |
| Terbutaline | R03AC03 | 0 |  |  |  | 0 | 0 |  | 0 |
| Teriparatide | H05AA02 |  |  |  |  |  |  |  |  |
| Testosterone | G03BA03 |  |  |  |  |  | 0 |  |  |
| Tetracycline | A01AB13 | 0 |  |  |  |  |  |  |  |
| Thiamazole | H03BB02 |  |  |  |  |  |  |  |  |
| Thiamine | A11DA01 | 0 |  |  |  |  |  |  |  |
| Tiaprofenic | M01AE11 |  |  |  |  |  | 0 |  |  |
| Tibolone | G03CX01 |  |  |  |  |  | 0 |  |  |
| Ticlopidine | B01AC05 | 0 |  |  |  |  |  |  |  |
| Lactase; Tilactase | A09AA04 | 0 |  |  |  |  | 0 |  |  |
| Tilidine/Naloxone | N02AX51 |  |  |  |  |  |  |  |  |
| Timolol | C07AA06 | 0 |  |  |  | 0 | 0 |  | 0 |
| Tolbutamide | A10BB03 | 0 |  |  |  |  |  |  |  |
| Tolcapone | N04BX01 | 0 |  |  |  | 0 |  |  |  |
| Topiramate | N03AX11 | 0 |  |  |  |  |  | 0 | 0 or 1 |
| Torsemide | C03CA04 | 0 |  |  |  |  |  |  |  |
| Travoprost | S01EE04 |  |  |  |  |  | 0 |  |  |
| Trimethoprim | J01EA01 | 0 |  |  |  |  | 0 |  | 0 |
| Trypsin | B06AA07 | 0 |  |  |  |  |  |  |  |
| Tuberculin Purified Protein Derivate | V04CF01 | 0 |  |  |  |  |  |  |  |
| Ubidecarenone | C01EB09 |  |  |  |  |  | 0 |  |  |
| Ursodiol | A05AA02 | 0 |  |  |  |  |  |  |  |
| Valsartan | C09CA03 | 0 |  |  |  |  |  |  |  |
| Vardenafil | G04BE09 |  |  |  |  |  | 0 |  |  |
| Varenicline Tartrate | N07BA03 |  |  |  |  |  | 0 |  |  |
| Vecuronium | M03AC03 | 0 |  |  |  |  |  |  |  |
| Vemurafenib | L01XE15 |  |  |  |  |  |  |  |  |
| Verapamil | C08DA01 | 0 |  |  |  | 0 | 0 |  | 0 |
| Vitamin E | - | 0 |  |  |  |  |  |  |  |
| Zaleplon | N05CF03 | 0 |  |  |  |  |  |  |  |
| Zinc Gluconate | A12CB02 | 0 |  |  |  |  |  |  |  |
| Zinc Sulfate | A12CB01 | 0 |  |  |  |  |  |  |  |
| Zolpidem | N05CF02 | 0 |  |  |  |  | 0 |  | 0 |
| Zopiclone | N05CF01 | 0 |  |  |  | 0 | 0 |  | 0 |

References

1. Carnahan RM, Lund BC, Perry PJ, Pollock BG, Culp KR: **The Anticholinergic Drug Scale as a measure of drug-related anticholinergic burden: associations with serum anticholinergic activity**. *J Clin Pharmacol* 2006, **46**(12):1481-1486.

2. Ancelin ML, Artero S, Portet F, Dupuy AM, Touchon J, Ritchie K: **Non-degenerative mild cognitive impairment in elderly people and use of anticholinergic drugs: longitudinal cohort study**. *BMJ* 2006, **332**(7539):455-459.

3. Rudolph JL, Salow MJ, Angelini MC, McGlinchey RE: **The anticholinergic risk scale and anticholinergic adverse effects in older persons**. *Arch Intern Med* 2008, **168**(5):508-513.

4. Han L, Agostini JV, Allore HG: **Cumulative anticholinergic exposure is associated with poor memory and executive function in older men**. *J Am Geriatr Soc* 2008, **56**(12):2203-2210.

5. Ehrt U, Broich K, Larsen JP, Ballard C, Aarsland D: **Use of drugs with anticholinergic effect and impact on cognition in Parkinson's disease: a cohort study**. *J Neurol Neurosurg Psychiatry* 2010, **81**(2):160-165.

6. Sittironnarit G, Ames D, Bush AI, Faux N, Flicker L, Foster J, Hilmer S, Lautenschlager NT, Maruff P, Masters CL *et al*: **Effects of anticholinergic drugs on cognitive function in older Australians: results from the AIBL study**. *Dement Geriatr Cogn Disord* 2011, **31**(3):173-178.

7. Program AB: **Anticholinergic Cognitive Burden Scale 2012 Update**. 2012. https://www.uea.ac.uk/documents/3306616/10940915/Anticholinergics/088bb9e6-3ee2-4b75-b8ceb2d59dc538c2.

8. Duran CE, Azermai M, Vander Stichele RH: **Systematic review of anticholinergic risk scales in older adults**. *Eur J Clin Pharmacol* 2013, **69**(7):1485-1496.
